# Study on rheological properties of composite propellant slurry in the mixing process by using experimental and numerical simulation†

**DOI:** 10.1039/d4ra05964f

**Published:** 2024-12-02

**Authors:** Zhiming Guo, Xiaolong Fu, Riccardo Rossi

**Affiliations:** a Departament d'Enginyeria Civil i Ambiental (DECA), Universitat Politécnica de Catalunya (UPC) Jordi Girona 1 Barcelona 08034 Spain zhiming.guo@upc.edu riccardo.rossi@upc.edu; b School of Mechatronics Engineering, North University of China Taiyuan 030051 PR China sedisim@nuc.edu.cn; c Xi'an Modern Chemistry Research Institute Xi'an 710065 China

## Abstract

Researchers have extensively focused on the safety of the solid propellant preparation process, particularly the mixing process that was deemed critical for the safety of the entire procedure. Herein, the rheological curves of propellants at different stages of mixing are obtained experimentally. The obtained curves are fitted using the Herschel–Bulkley non-Newtonian model. CFD calculations were performed using this data, allowing us to obtain the pressure and velocity evolution within the fluid domain during mixing. Such calculations are then used to assess the variation in the homogeneity of the mixture over time. Experimental evidence reveals that the viscosity of the composite propellant slurry is 32 Pa s after the addition of fine-grained ammonium perchlorate (AP). The propellant slurry tends to be 15 Pa s at the end of mixing. Both the morphology and elemental analysis of the slurry demonstrated that the fine AP is more distributed near the coarse AP. In contrast, the aluminum powder is distributed more evenly within the propellant matrix. Moreover, theoretical simulation input parameters are obtained using the Herschel–Bulkley model fitting. Numerical simulation results show that the paddle and the inner wall are more prone to a sudden increase in pressure and velocity concentration due to the shear effect, and the mixing homogeneity and safety analysis of the propellant slurry can be visualized in a short time period.

## Abstract 2

This study explores rheological properties related to propellant slurries from the standpoint of experiments and simulations.

## Graphical abstract

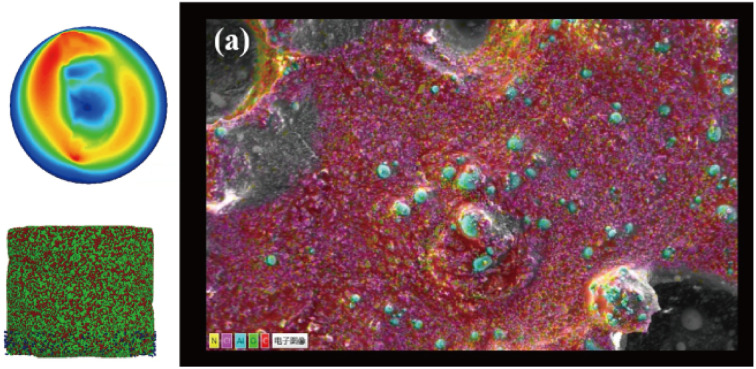

## 1. Introduction

Composite solid propellants contain 70–80% of oxidizers such as ammonium perchlorate (AP), 10–15% of powdery metallic fuel such as aluminum, and polymeric binders such as hydroxyl-terminated polybutadiene (HTPB),^1^ which play an important role in the military and aerospace fields as a working substance for rocket or missile engines. Typically, composite solid propellants are prepared and manufactured using vertical mixing machines by capitalizing on their benefits of uniform mixing and efficient production capabilities.^2^ In the manufacturing of solid propellants, the mixing process is the most important and dangerous process. Mixing could result in combustion, explosions, and other safety concerns. Numerical simulation plays a significant role in ensuring the safety of solid propellant preparation.

The simulation parameters for the mixing process are derived from the propellant's rheological properties. The main factors affecting the rheological properties of highly filled composites include particle size distribution, particle nature and shape, particle interactions, maximum stacking fraction, and matrix viscosity, with matrix viscosity being a key parameter.^3,4^ The rheological properties of the propellant slurry mainly involve yield value, the relationship between shear stress, apparent viscosity and shear rate, and constitutive model.^5^ The propellant slurries behave as pseudoplastic fluids in a certain shear rate range.^5,6^ Restasari *et al.*^7^ investigated the pseudoplasticity of propellant slurry with varying aluminium content for castability development. The results show that the more aluminium contents, the higher the viscosity and the more pseudoplastic the propellant slurry. The slurry of 18% aluminium is characterized by high viscosity and pseudoplasticity. Chauhan *et al.*^8^ proposed that processing at 45 °C gave lower viscosity and better pot life than 40 °C. Addition of some extra percentages of plasticizer (DOA) helps lower the end of mix viscosity and extends the pot life. The Herschel–Bulkley empirical model describes the viscosity law that governs solid propellant shear deformation.^9,10^ Due to the fact that the Herschel–Bulkley model incorporates the effect of yield stress on AP-based composite propellant,^11,12^ a very close fit can be made. The continuous evolution of the material properties during the mixing is not included in the work due to the lack of available data to characterize such behavior. Instead, different values of the Herschel–Bulkley parameters are considered for the different stages of filling (that is, when a new component is added).

Previous studies have highlighted the importance of cross-validation with computational fluid dynamics (CFD) models based on experimental data.^13^ CFD is a powerful tool that provides relevant process information and evaluates stirred vessels.

Researchers use CFD technology to simulate fluid movement in stirred containers.^14–16^ 3D numerical simulation of the shear thinning fluids in a twin-sigma blade mixer was performed using the finite element method (FEM) combined with the mesh superimposition technique as implemented by Polyflow. The center zone of the mixer between the two blades has excellent distributive and dispersive mixing ability, with high shear rates and mixing-index values.^17^ Previous studies have focused on the kneading parameters of twin-blade planetary mixers for highly viscous fluids.^18–20^ Although extensive research has been carried out on the design and motion of vertical kneaders, there have been few in-depth studies on the rheological properties of composite solid propellants and the simulation of the mixing process. Numerical simulation is a critical step in establishing a theoretical basis for the adjustment of process parameters to ensure safe production by providing a theoretical basis for adjustment.

In this paper, the mixing process of slurry in the mixing kettle of a twin-blade planetary kneader is the research object. The flow characteristics of slurry in the mixing kettle are studied using the finite element analysis method. The rheological properties of the slurry were experimentally calibrated to determine the viscosity characteristics at different stages during the slurry mixing process, and the chaotic characteristics of the solid propellant slurry fluid dynamics system in the mixing tank were analyzed through the established numerical model simulation, revealing the solid propellant slurry flow laws in complex mixing domains. Moreover, scanning electron microscopy (SEM) experiments were used to analyze the solid particle distribution at the cross-section after the propellant slurry was mixed, and the degree of deviation between the measured value and the calculated value of the uniformity of the solid propellant slurry was calculated, and the evaluation standard of slurry mixture uniformity was obtained. It is hoped that the results of this study will provide theoretical support for safety research on solid propellant slurry mixing processes as well as performance analyses of mixing processes.

## 2. Methodologies

### 2.1 Experiments

#### 2.1.1 Materials

Hydroxy-terminated polybutadiene (HTPB) as a binder is manufactured by free radical polymerization with a molecular mass of 2300–2800 g mol^−1^, hydroxyl value = 47.1 mg KOH per g, polydispersity = 2.5, viscosity at 30 °C = 2000–3000 cP, density = 0.8–0.9 g cm^−3^. Ammonium perchlorate (AP) as an oxidizer was used in two-fold distributions having average particle sizes of 120 μm and 7 μm, respectively, with a density value of 1.95 g cm^−3^. Dioctyl sebacate (DOS) with a density value of 0.926 g cm^−3^, toluene diisocyanate (TDI) with a density of 1.225 g cm^−3^, aluminum powder (Al) with a mean diameter of 5 μm. Table 1 provides information about the composition of the composite propellant.

**Table 1:** The propellant composition

Composition	State	Parameter	Content (%)
Ammonium perchlorate (AP)	Solid	Particle size (coarse): 100–140 μm	60
		Particle size (fine): 6–8 μm	
Al	Solid	Particle size: 29 μm	18
HTPB	Liquid	—	14.1
DOS	Liquid	—	7
TDI	Liquid	—	0.9

#### 2.1.2 Propellant slurry preparation

The propellant slurry was processed in a vertical planetary kneader (5 L maximum capacity) with a solid loading of 78% consisting of aluminium powder and AP particles with a coarse-to-fine ratio of 2 : 1. The remaining 22% is liquid materials (HTPB, DOS and TDI). This mixer features a double-blade system with a rotational speed of 30 rpm. Utilizing a jacketed hot water circulation system, the slurry temperature is maintained between 50 and 55 °C.

**Fig. 1:**
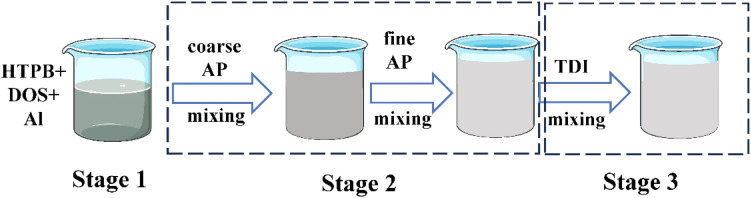
Mixing process of propellant slurry.

The mixing process was done in several steps. A 10 min mixing of all liquids and Al powder was conducted, except for the curing agent TDI, which is the curing agent. The second was the addition of coarse AP particles and fine AP particles, respectively, for a period of 10 min each. After that, the slurry was mixed for 40 min under vacuum. In the end, TDI was added and mixed under atmospheric and vacuum pressure for a period of 10 and 15 min, respectively (Fig. 1).

#### 2.1.3 Rheological properties experiment

During this mixing process, fluid properties are changed. To understand the fluid properties at each stage, the liquid's rheological properties at stages 1, 2, and 3 are monitored using a rheometer. The test was performed using a HAKKE RS300 rotational rheometer with a shear rate of 0–15 s^−1^, 30 steps, and a temperature setting of 50 °C. Fig. S1† shows the rheometer. As a result of fitting the model data, the slurry in the first stage was modeled as a Newtonian fluid, with viscosity independent of shear rate, whereas the second and third stages were modeled using the Herschel–Bulkley model.

The Herschel–Bulkley (H–B) model characterized the rheological profile of pharmaceutical pastes and H–B models usually have high fitting coefficients. The propellant rheological results were fitted to the Herschel–Bulkley equation to determine the pseudoplasticity index *n*, the yield stress *τ*_0_, and the consistency value of *K*. The Herschel–Bulkley equations were achieved after the fundamental rheological parameters of the Herschel–Bulkley model were determined.^21,22^ The Herschel–Bulkley constitutive model characterizes fluid properties as follows: the fluid exhibits a yield value and flow does not occur if the fluid shear stress is below this yield value. According to the H–B model, viscosity can be expressed in the following manner:1
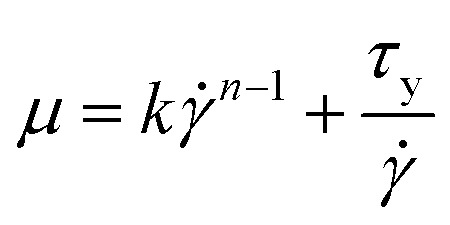
in the formula, *μ* is the dynamic viscosity of the fluid, *k* is the consistency index, *τ*_y_ is the yield stress, *γ̇* is the shear rate, and *n* is the control parameter.

#### 2.1.4 Measurement of homogeneity

This study employs SEM (Quanta FEG 600) to intuitively track HTPB propellant's mesostructure evolution during mixing. To observe the distribution of solid particles more clearly, a mapping element scanning analysis was performed. Based on the distribution of the N element in the propellant slurry, it can be determined if the solid filler has a significant effect on mixing.

### 2.2 Simulation details

#### 2.2.1 Geometry model of the kneader

Vertical Kneader is widely used in the mixing process of propellant slurry due to its advantages of mixing uniformity and high efficiency. The mathematical model of the twin-blade planetary mixers was first developed by Wang *et al.*^19^ in 1993. The establishment of the mathematical model provides the foundation for computer-aided design (CAD) design and numerical simulation of the twin-blade planetary mixers. Fig. S2† shows the twin-blade planetary kneader model in this study, which consists of a hollow blade and a solid blade. There are mathematical equations that describe the trajectory of both hollow and solid paddles, and these equations are presented in eqn (2) and (3).2
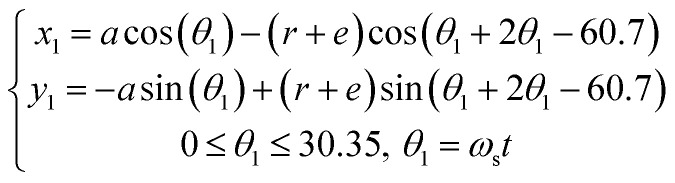
3
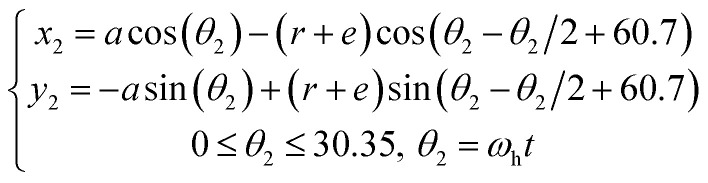
where *a* is the distance between the centres of the two mixing paddles. In mixing paddles, *r* is the radius, *ω*_s_ is the angular velocity of the solid paddle, and *ω*_h_ is the angular velocity of the hollow paddle.

The vessel diameter is *D* = 540 mm. The diameter of the two blades is *d* = 55 mm. The stirring paddle's inner diameter is *d* = 270 mm. The minimum clearance is *e* (which is equal to 3 mm). The height of the stirring paddle is *h* = 405 mm. The kneader's kneading regions consist of the blade–blade, blade–wall, and blade–bottom kneading regions.

Furthermore, the process of simulating the motion of the stirring paddle blades in the fluid must be considered. The motion of solid and hollow blades can be considered in rotation and revolution. The revolution part represents the rotation of the hollow blade around the *Z*-axis of the coordinate system, while the rotational part represents the rotation of the hollow blade around its own axis. The system of two blades rotates with respect to a common center of rotation with a different rotation speed. *N*_G_, *N*_H_ and *N*_S_ are the global speed, the rotation speed of the hollow blade and the solid blade, respectively. Experimentally, the rotational speed of the kneader was set to 30 rpm, the rotational speed of the hollow paddle was set to 10 rpm, and the rotational speed of the solid paddle was set to −5 rpm (“+” and “−” represent the opposite directions of motion).

#### 2.2.2 Initial assumptions

The process to be simulated is the mixing of multiple materials under vacuum conditions. The material is stacked in segregated layers, with a liquid component at the bottom and a powder with varying diameters on the top. The objective is to evaluate the physical mixing between the different components of the mixture. Consequently, as a simplification, the chemical reaction between the different components is not considered during the simulation. Furthermore, it is agreed that the simulated material can be treated as a single fluid with rheology described by the Herschel–Bulkley constitutive law model. Under this assumption, the entire material body behaves the same independently of the specific distribution of material within the volume. Since the slurry behavior is considered independent of the exact distribution of material, such an assumption has a critical advantage. Simulating multiple species can be divided into two stages to obtain accurate statistics: a first stage for non-Newtonian single-phase flows and a second for mixing between species. A variational multi-scale method is used for fluid domain discretization. The mixer surface is superimposed and implicitly positioned on the top of the fluid domain by a level-set approach. Its movement is coupled to the fluid domain by a variation of the cut-FEM approach. It is further observed that the fluid solution is periodic in time. This indicates that simulating just one period can be later applied to simulating multiple periods of particle mixing. The solver comprises two stages, one for the fluid and one for the particles, with the second stage building on the results obtained during the first stage.

#### 2.2.3 Fluid solver

The solver discretizes the incompressible Navier–Stokes equations described by the partial differential equations (PDEs):4
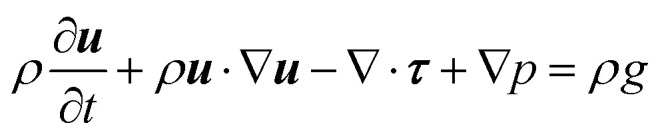

5 ∇·***u*** = 0

where *ρ* is the density, ***u*** is the velocity vector, *p* is the pressure and *g* is the gravity acceleration. The term ***τ***(***u***) is the shearing component of the Cauchy stress tensor, such that *σ* = *τ* + *pI*. Such description covers both Newtonian and non-Newtonian fluids. Specifically, a non-Newtonian, Herschel–Bulkley approach is employed in all of the simulations performed. This corresponds to assuming a constitutive behaviour of the type:

6 ***τ*** = 2μ*∇^s^***u***

where the nonlinear secant viscosity μ* is defined as eqn (7).7
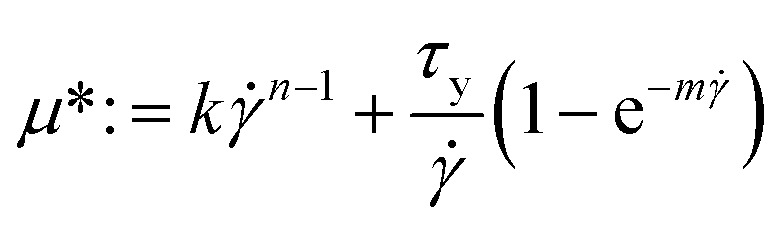
With 
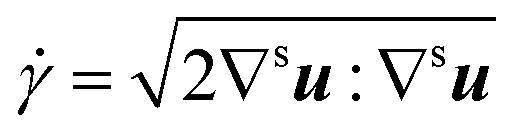
 and where *k* is the shear viscosity, *τ*_y_ is the yield stress, *γ̇* is the shear rate, *n* and *m* are the control parameters. *k*, *n* and *τ*_y_ were obtained from rheological profile by fitting the Herschel–Bulkley model. High values of such parameters imply converging closely to the original model. As observed in the result section, alternative choices were possible for the non-Newtonian law; however, the model chosen appears to represent closely the experimental behaviour and is thus deemed adequate for the solution.

#### 2.2.4 Particle tracking

Throughout the whole project, particles are exclusively considered as trackers, indicating that they are transported with the flow and present no interaction with neighbouring particles, thus serving exclusively to trace where different types of materials are after the mixing takes place. To this end, every particle knows its current position in space. Tracker particles are moved employing a fourth-order Runge–Kutta approach to minimize errors in the integration of the trajectories. This corresponds to the following five-step integration process:

8 ***u***_1_^*p*^ = Δ*t****u***(*x*_*n*_,*t*_*n*_)

9
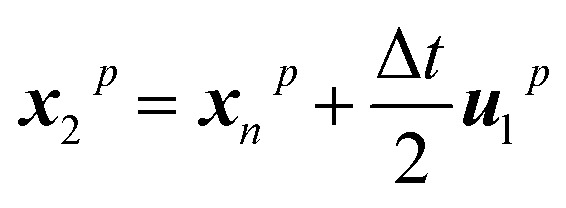
10
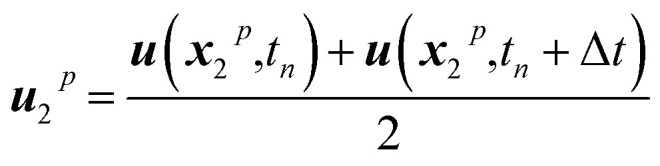
11
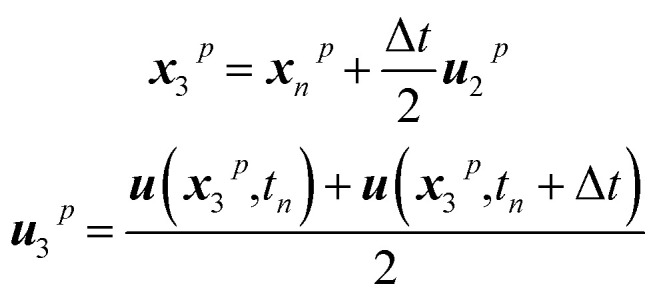
12
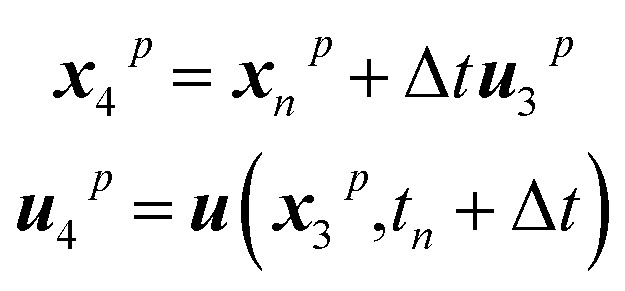
13
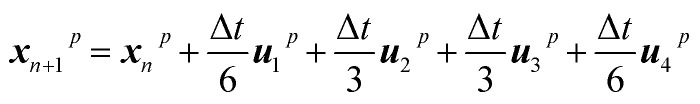
where ***x***_*n*_p is the position of a given particle at the time step *n* and ***u***(*x*_2_^*p*^,*t*_*n*_) is the velocity as found at the position ***x***_2_^*p*^ when interpolating the Eulerian velocity field, and the time increment Δ*t* is 0.05 s.

The use of such high-order methods allows for tracking the motion of the particles over thousands of revolution cycles and thus to model the entire mixing process. Statistics about the evolution of the tracer positions in space are obtained by subdividing the space into 50 horizontal layers (a user-defined choice) and by measuring the number of tracing particles that fall within each of such layers. This count allows reconstructing the spatial concentration of the different components in the mixture within the layers, thus allowing to evaluate the spatial uniformity of the mixing. A synthetic description of the mixture quality is presented here in terms of the Kramer mixing index^23^ (a value of 1 represents perfect mixing, meaning that the statistical distribution of particles representing different species is the same all over the volume). No error evaluation has been performed to assess the quality of the simulation as the work employed the finest mesh that was affordable given the computing resources and the available computing time. The Kratos code has, however, been verified in application to similar problems in the context of various research works example,^24,25^ which let us believe that the results are at a minimum qualitatively correct.

#### 2.2.5 Simulation process

All simulations were done using the Software Kratos Multiphysics,^26^ employing a finite element-based incompressible fluid solver. The solver is based on the monolithic solution of the velocity–pressure pairs and is stabilized using a VMS approach.^27^ Strict bounds were prescribed for the convergence of both the velocity and pressure fields 
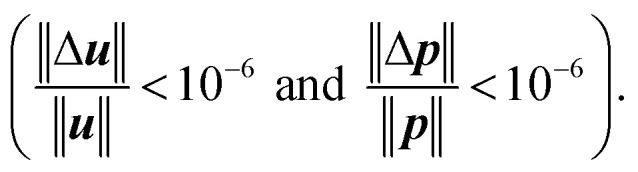
 The complete form (based on ∇^*s*^***u***) of the viscous term is employed instead of the Laplacian approximation to guarantee an accurate solution of the non-Newtonian rheology.^24^ The motion of the mixer blades is taken into account by the use of an immersed CFD approach.^25^ A voxel-based search structure is employed to maximize the spatial searches required for the motion of particles and gather the statistical results. The gathering of all of the statistical data, as well as the calculation of aggregated indices, is managed through the Kratos python-level API by taking benefit of the numpy library.^28^

The simulation is based on the following stages (to be repeated as needed as new components are aggregated to the mixture):

(1) Generate a random distribution of tracing particles identifying the different materials to be mixed. Particles are pseudorandomly seeded within the volumes occupied originally by the different species. A total of around 1 M tracing particles are seeded in the volume to ensure good statistical representativity.

(2) Perform a CFD calculation, considering the motion of the mixing device by the use of an immersed approach. The mixture behaviour is considered non-Newtonian using the identified material properties. The simulation must be carried out to the point at which the effect of mixing initialization becomes negligible and the flow pattern become cyclical.

As a post process:

(3) Track the motion of particles over several cycles (note that CFD results in terms of velocities are reused to allow the simulation of a longer time span as may be needed to characterize the entire mixing process).

(4) At every time step evaluate the distribution of the different species as a function of the layer (the entire mixer is divided into vertical layers used as buckets in sampling the number of particles belonging to the different species).

(5) Postprocess the results obtained in 4 to compute the mixing index (Kramer index is used in this work. Alternative indices, such as the Lace *y* index could be considered with relative ease).

## 3. Results and discussion

### 3.1 Rheological properties

**Fig. 2:**
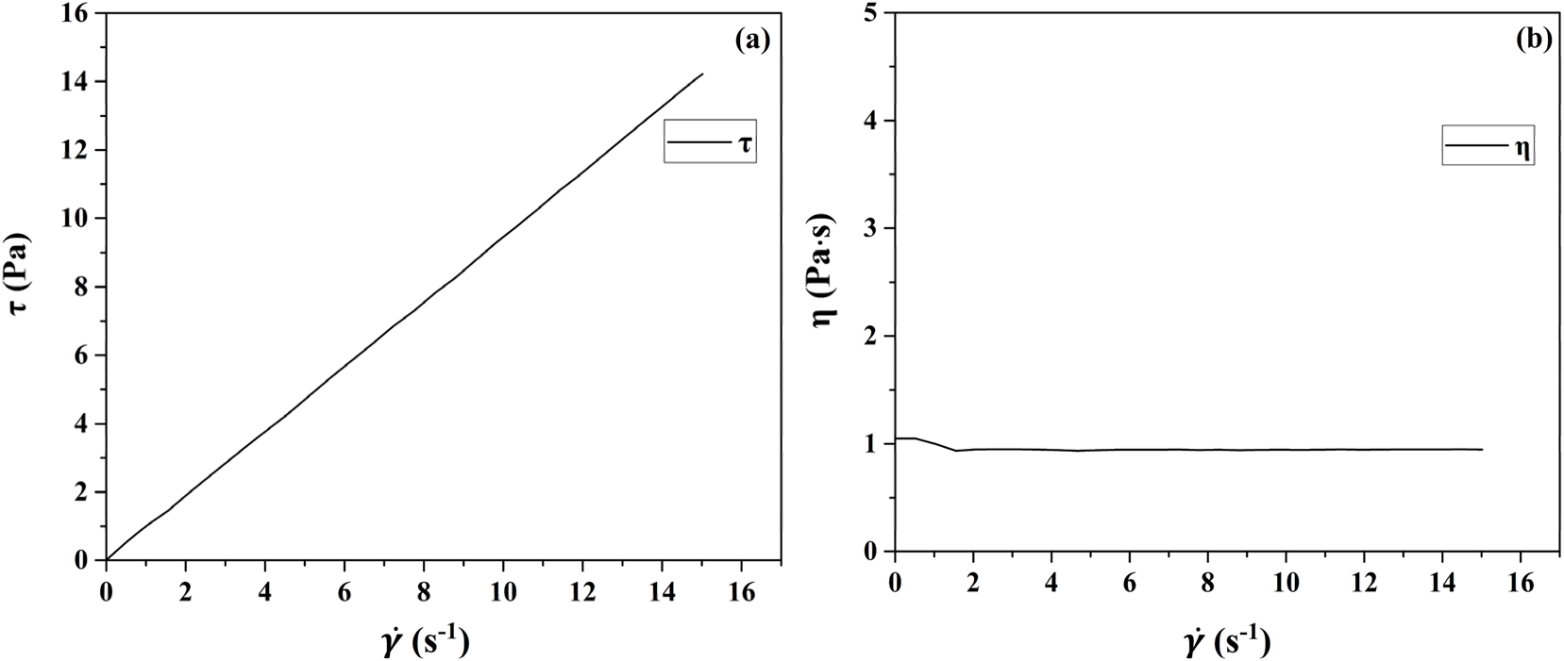
Rheology curve of stage 1.

**Fig. 3:**
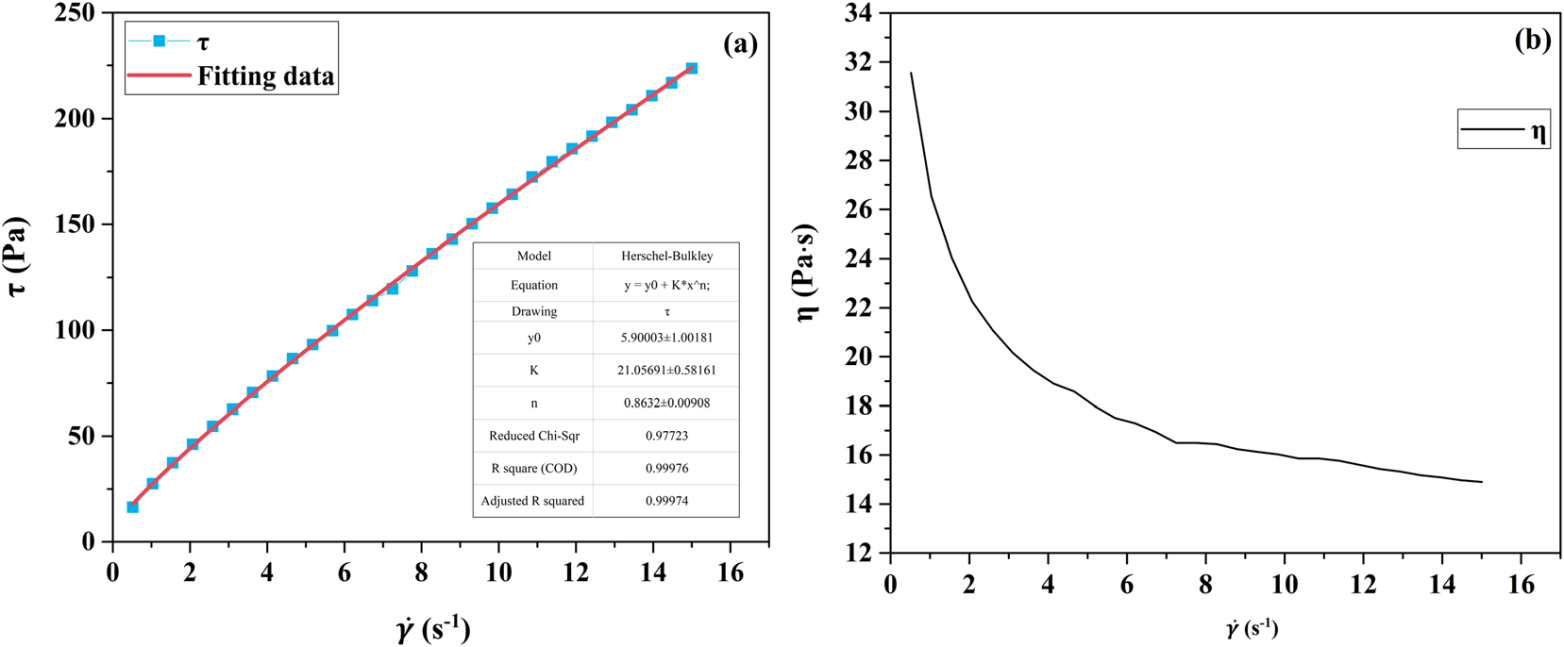
The curves of shear stress (a) and viscosity (b) *versus* shear rate.

The rheological profiles of the propellant slurry at each stage of the mixing process were tested using a HAKKE RS300 rotational rheometer, as shown in Fig. 2 and 3. At very low and high shear rate,^4^ viscosity exhibits Newtonian behavior. The rheological profile of the slurry after stirring in the first stage has Newtonian fluid properties. Its viscosity does not change with time and remains constant at 0.95 Pa s. The rheological curve of the slurry after mixing homogeneously in the second stage is characterized by non-Newtonian fluid characteristics, exhibiting shear thinning phenomena. This shear-thinning behavior indicates the pseudo-plastic characteristics of slurries.^29^ The slurry viscosity increased when coarse-grained AP was added, with a maximum viscosity of 32 Pa s. In the process of mixing and homogenization, the viscosity of the slurry is gradually reduced by the shearing effect of the mixing paddle. At the end of the second stage, the viscosity is about 15 Pa s.

The first stage slurry is a Newtonian fluid whose viscosity does not vary with shear rate. The second stage was fitted using the Herschel–Bulkley model, and the fit matched the experimental data. Fig. 3(a) shows the second mixing stage fitting curve. Where *K* = 21, *n* = 0.8632, and yield stress = 5.9. To determine the input parameters for the simulation model, *k* and *n* were determined by fitting the Herschel–Bulkley model.

### 3.2 Pressure and velocity distribution during mixing

**Fig. 4:**
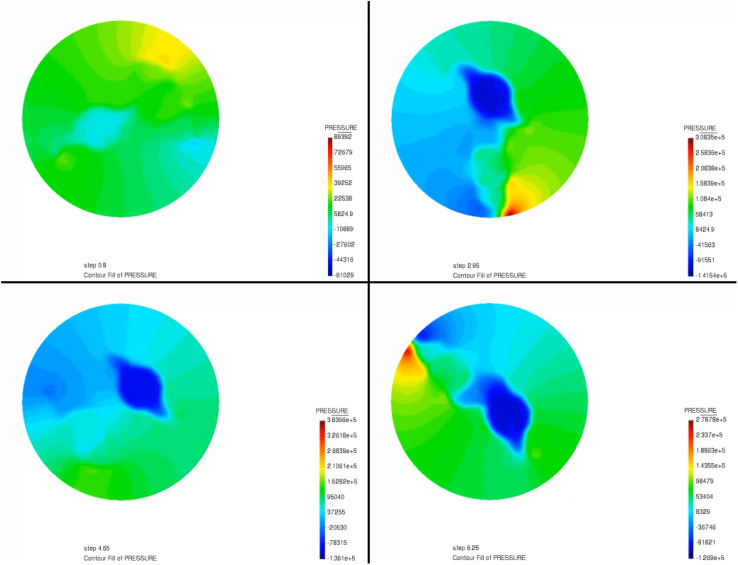
The pressure field distribution.

**Fig. 5:**
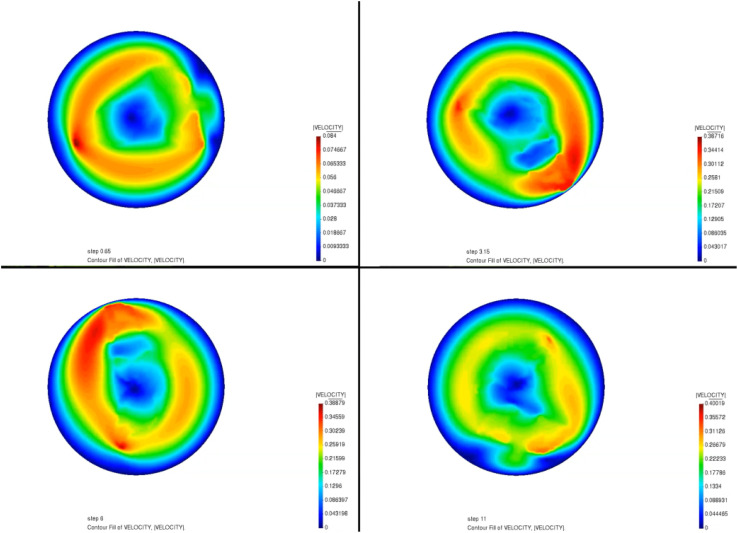
The velocity field distribution.

The mixer itself is composed of two blades each rotating with respect to its own axis at a specific rotation speed. Fig. 4 and 5 display the pressure and velocity distribution of mixing as the paddle rotates during the mixing process. As can be seen in Fig. 4, before mixing, the pressure appears in green, indicating that the pressure is low throughout the region at this time. With rapid mixing, the pressure in the edge area (for the kneading pot is the pot wall) appeared at the highest point because when the paddle is closer to the inner wall, the tip of the paddle is subjected to the greatest pressure. This indicates that as the gap between the paddle and the inner wall decreases, the shear effect of the propellant slurry is more significant. This is mainly when the hollow paddle is closest to the inner wall, the hollow paddle tip of the linear velocity is the largest; moreover, when the material is subjected to the largest shear, the paddle and the inner wall of the smallest gap will strengthen the extrusion of the material such that the material in the hollow paddle produces a reaction force and the hollow paddle is subjected to the highest pressure. Fig. 5 shows the flow velocity field during mixing. It can be seen in Fig. 5 that the fluid rotates with the action of the paddles as the paddles rotate. There is inhomogeneity in the fluid velocity close to the paddles, and it is more uniform away from the paddles. It has the highest velocity at the edges of the paddles and high velocity for mixing the slurry in its vicinity. In contrast, the velocity distribution is lower at locations temporarily out of reach of the paddles. A parametric model was used to study the mixing of composite propellants in a vertical kneader at a rotation speed of 30 rpm. It has been determined that propellant slurry is more likely to accumulate between the paddle and the inner wall when pressure is built up and velocity is concentrated. This has been a particularly dangerous situation.

### 3.3 Mixing process simulation and analysis

**Fig. 6:**
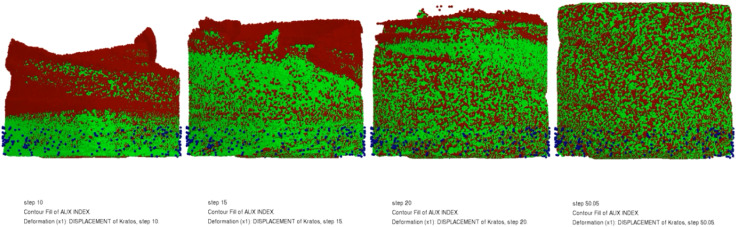
The homogeneity changes of the propellant slurry during mixing simulation.

**Fig. 7:**
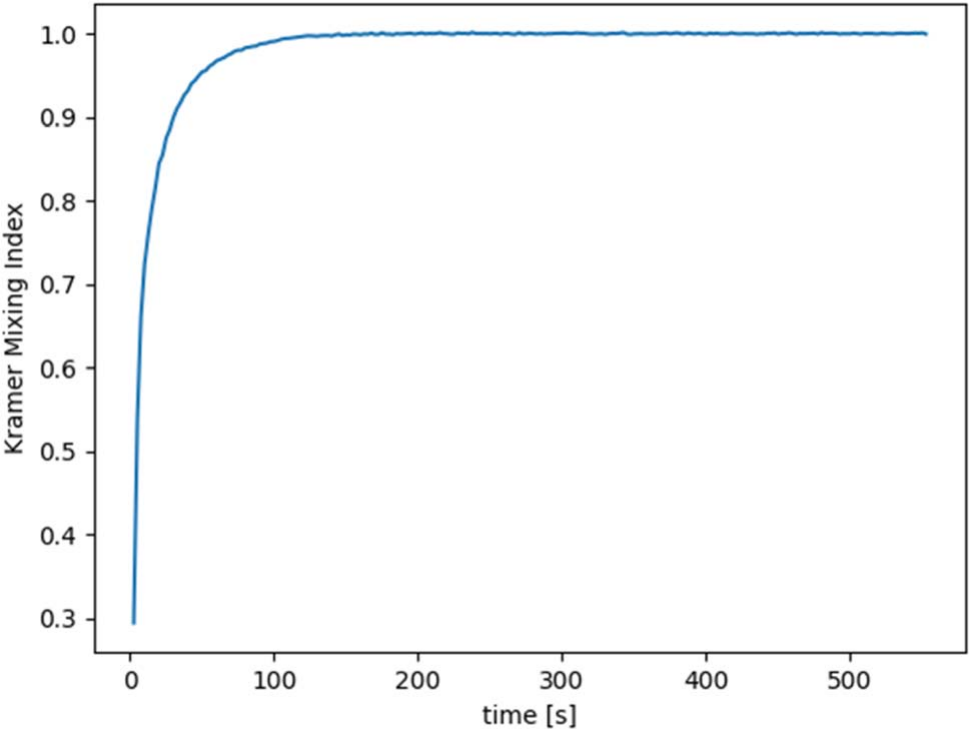
The homogeneity changes curve of propellant slurry during mixing simulation.

Based on the simulation of the propellant mixing process, Fig. 6 shows how the uniformity variation of the propellant slurry fluid can be visualized. The green part can be considered the premix of the propellant liquid phase component and the aluminum powder. The red part is the AP. As the mixing proceeds, the red part gradually mixes well with the green part, which reproduces the pharmaceutical slurry mixing process. As shown in Fig. 7, the uniformity change curve of the propellant slurry during the simulated mixing process. It has been demonstrated that by using simulation, the uniformity index may be achieved within 200 s depending on the amount of slurry used.

### 3.4 Mixing homogeneity analysis

**Fig. 8:**
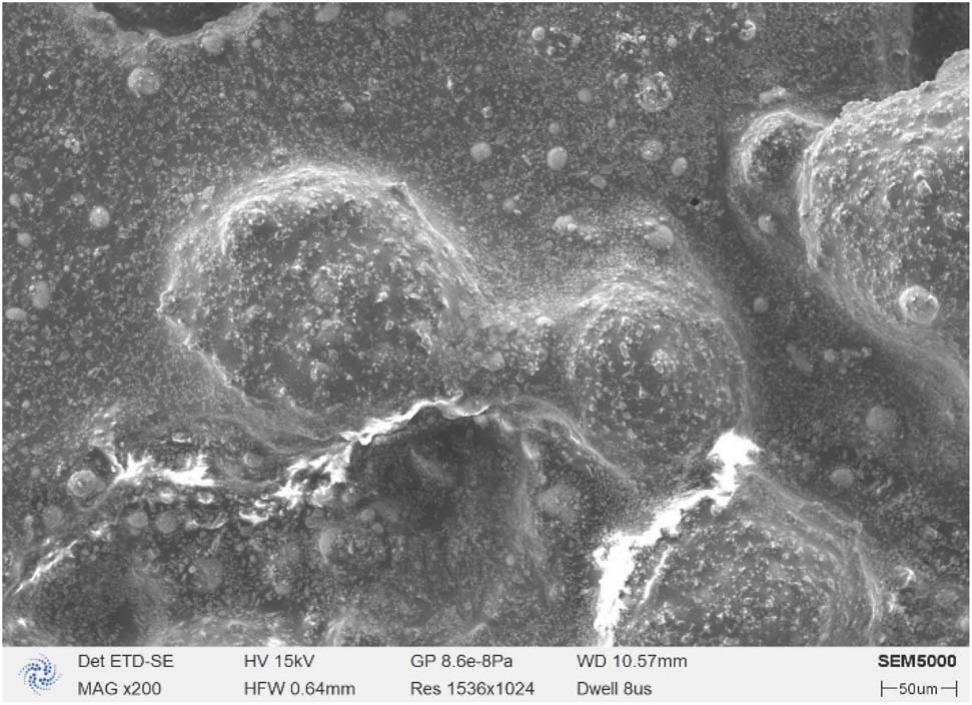
Scanning electron micrographs of the propellant slurry.

**Fig. 9:**
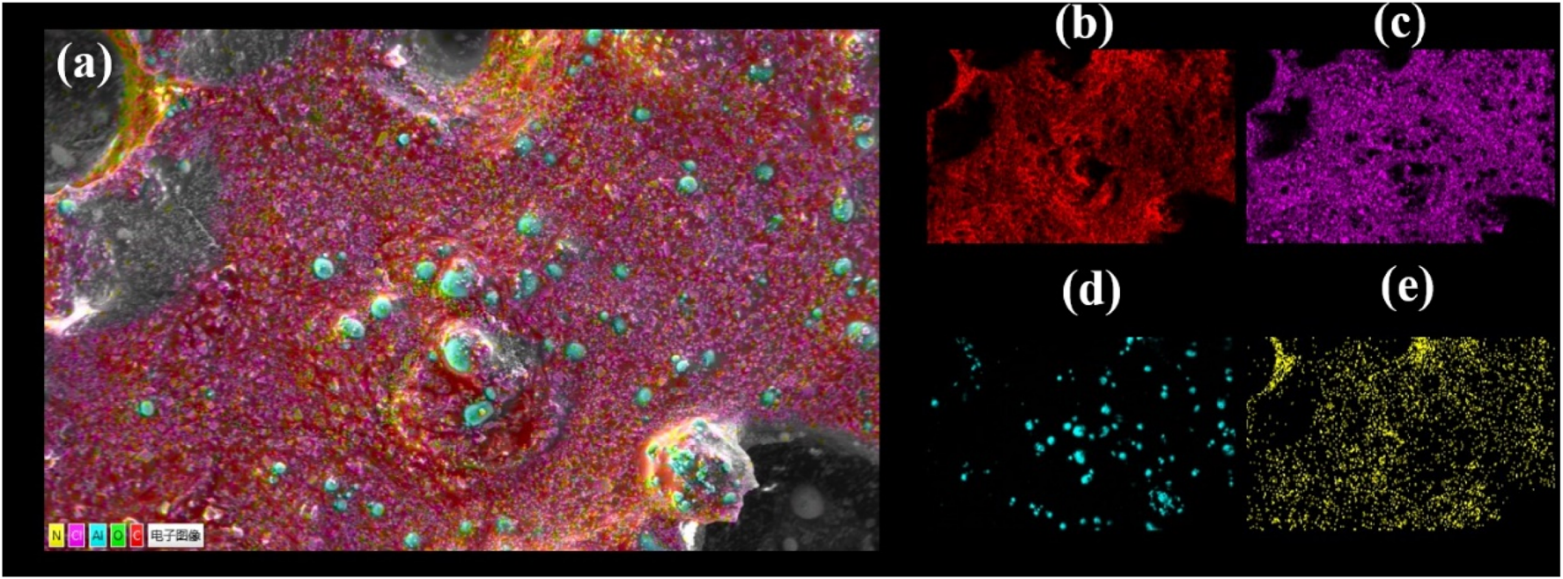
The EDS spectra of propellant slurry (a), C (b), Cl (c), Al (d), N (e).

To study the homogeneity of the propellant slurry after mixing, the morphology and elements of the slurry were analyzed by scanning electron microscopy combined with EDS analysis. The results are shown in Fig. 8. The few large particles around 100 μm are AP, the fine-grained AP is partly attached to the large AP particles and partly uniformly distributed in the propellant slurry, and the spherical solid is aluminium powder. Further, the elemental distribution of the propellant slurry was scanned, and the results are shown in Fig. 9. N and Cl distributions indicate that the AP was well-mixed, whereas the Al powder distributions indicate that the Al powder was distributed sporadically due to its low concentration.

## 4. Conclusion

In this paper, the propellant slurry mixing process has been studied by experiment and simulation, and the main conclusions are as follows:

(1) The rheological profile of propellant slurry was tested. The key input parameters for the simulation were obtained by applying the Herschel–Bulkley model fitting, where *K* = 21, *n* = 0.8632, and yield stress = 5.9.

(2) A simulation of the propellant mixing process was carried out to obtain the pressure and velocity distributions of the simulation process, and the results showed that the propellant is most likely to have pressure buildup and velocity concentration between the paddle and the inner wall, where this station is also the most dangerous. Through the propellant slurry mixing process simulation, the uniformity change of the slurry can be visualized in a short period of time.

(3) SEM and EDS analyses were used to analyze the morphology and elemental distribution of the mixed slurry. The results of this study showed that spheres of aluminum powder and fine aluminum powder were dispersed in the matrix of the slurry and that the fine aluminum powder adhered better to the large particles.

## Data availability

The data supporting this article have been included as part of the ESI.†

## Conflicts of interest

The authors declare that they have no known competing financial interests or personal relationships that could have appeared to influence the work reported in this paper.

## Supplementary Material

RA-014-D4RA05964F-s001
