# Supplementary material for: Study on rheological properties of composite propellant slurry in the mixing process by using experimental and numerical simulation
Source: RSC Adv. 2024 Dec 2;14(51):38162–70. doi: 10.1039/d4ra05964f (PMC11609947; doi:10.1039/d4ra05964f)
Supplement: RA-014-D4RA05964F-s001 [file RA-014-D4RA05964F-s001.pdf]

Supplementary Information (SI) for RSC Advances.
This journal is © The Royal Society of Chemistry 2024

Electronic Supplementary Information (ESI)

Study on rheological properties of composite propellant slurry in mixing process by using experimental and numerical simulation

Zhiming Guo[a,b], Xiaolong Fu[c], Riccardo Rossi*[a]

[a]*Departament d'Enginyeria Civil i Ambiental (DECA), Universitat Politécnica de Catalunya (UPC), Jordi Girona 1, Barcelona, 08034, Barcelona, Spain, E-mail address: zhiming.guo@upc.edu, riccardo.rossi@upc.edu*

[b]*School of Mechatronics Engineering, North University of China, Taiyuan 030051, PR China, E-mail address: sedisim@nuc.edu.cn*

[C] *Xi'an Modern Chemistry Research Institute, Xi'an, 710065, China.*

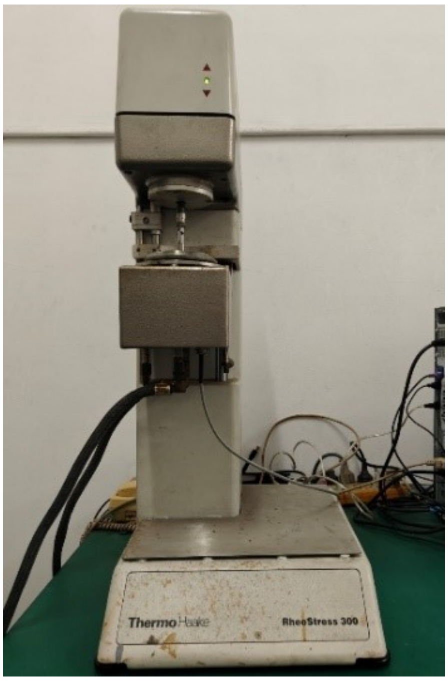

Fig. S1 HAKKE RS300 rotational rheometer.

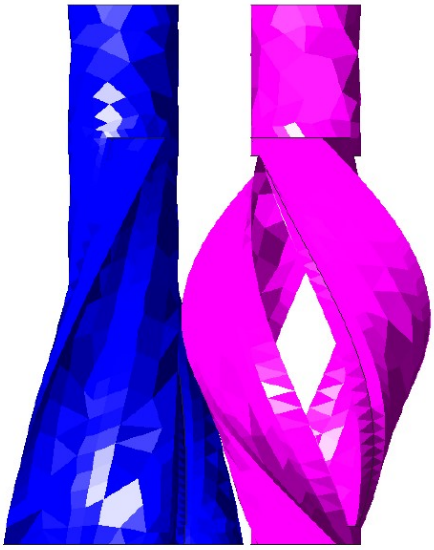

Fig. S2 Lateral view of the mixer.
